# Supplementary material for: Are carbon emissions trading and green financial instruments synergistic? -Comprehensive quantitative research based on content analysis
Source: PLoS One. 2024 Mar 7;19(3):e0298601. doi: 10.1371/journal.pone.0298601 (PMC10919601; doi:10.1371/journal.pone.0298601)
Supplement: S1 Data — (ZIP) [file pone.0298601.s001.zip › Data/地级市Z_F工作报告.zip!/║■─╧/╔█╤⌠╩╨/└┤╫╘╣1⁄2╓┌║┼í╛┬φ┐╦╩2╛▌═°í┐_2017.docx]

各位代表：

现在，我代表邵阳市人民政府，向大会报告政府工作，请予审议，并请各位政协委员和其他列席人员提出意见。

一、过去四年工作回顾

本届政府任期四年。这四年，面对增长速度换挡期、结构调整阵痛期、前期刺激消化期“三期叠加”，面对经济发展新常态和供给侧结构性改革，在党中央、国务院、省委、省政府和市委的坚强领导下，在市人大、市政协的监督支持下，我们高举加快发展旗帜，主动作为，稳中求进，统筹推进稳增长、促改革、调结构、惠民生、防风险各项工作，经济总体实力实现大跨越，跃居第二方阵湘中西部地区七市州首位；产业发展迈上新台阶，工业园区强势崛起，规模工业个数和增加值在全省排名前移二位，基本完成了工业化初期的光荣使命；基础设施明显改善，高速公路成倍增加，昂首迈入“高铁时代”，武冈机场即将试飞；城乡面貌焕然一新，挥手作别“脏乱差”，奋力改写“破旧矮”，美丽邵阳再不是昨日模样；民生福祉大改善，城乡居民收入跑赢GDP，增长速度连年稳居全省前三位。当本届政府即将结束任期之际，我们可以自豪地向各位报告：我们基本完成了市第十五届人民代表大会确定的目标任务，给全市人民交上了一份合格的答卷！

四年来，我们着力稳增长促发展，综合实力稳步跨越。全市主要经济指标大多保持两位数增长，增速高于全省平均水平，经济总体实力跃居全省第二方阵七市州首位。绩效考核连续四年获评全省先进，小康考核连续二年获评全省经济发展奖。地区生产总值由2012年的1028.4亿元增加到2016年的1520亿元（预计数，下同），年均增长9.6%，新增GDP过百亿元县（市、区）5个；财政总收入突破百亿大关，由85.04亿元增加到142亿元，年均增长13.47%，新增财政总收入过10亿元县2个；固定资产投资由761.6亿元增加到1635亿元，年均增长21%，四年累计完成投资5455亿元，是上一个五年的2倍；社会消费品零售总额由511.3亿元增加到835亿元，年均增长13%。全面小康建设总体实现程度由73.3%提升到87%，增幅全省第一。可以说，这四年，面对艰难的“下沉”压力，邵阳经济逆势上扬，“风景这边独好”！

四年来，我们着力建园区兴产业，发展后劲明显增强。大力推进新型工业化、新型城镇化和农业现代化。三次产业比由24.2：39.3：36.5调整为21.7：34.7：43.6，第三产业占比大幅提升。工业扩规升级。规模工业企业由760家增加到1135家，新增个数排全省第二。规模工业增加值年均增长10%。一批能源业、制造业、制鞋业等大型品牌企业相继落户邵阳。轻工、建材、食品、机械四大传统产业总产值突破千亿元大关，达到1546亿元。皮具箱包、发制品、打火机、小五金等传统特色产业增速明显，总产值达到375亿元。园区建设持续升温，县县都有一家省级工业园区。湘商产业园共完成固定资产投资105亿元，开工建设标准厂房422万平方米，竣工313万平方米，投产178万平方米，签约企业550家，入园企业290家，投产157家，实现产值48亿元，税收2亿元，带动就业2.6万人。园区产值在工业总产值中占比由51.7%上升到72%，提升幅度全省第三。农业稳步发展。粮食播种面积基本稳定，总产实现“四连增”。生猪、肉牛、奶牛出栏数和存栏数居全省首位。投资16.19亿元，实施农村土地整治项目154个、整治土地79.69万亩，隆回、新宁被评为全国国土资源节约集约模范县。建成油茶、奶业、药材等特色农业生产基地1190万亩，新增省级龙头企业19家。打响以城郊造林为重点的绿化战役，“三边三区三年”绿色行动造林50万亩，全市累计完成各类造林140.5万亩。第三产业蓬勃发展。新增限额以上商贸流通企业614家，总数达825家。金罗湾国际商贸城、湘西南农产品物流中心等20个商贸物流项目全面启动。旅游收入达721亿元，增加值占GDP比重由2.1%提高到2.9%。崀山成功创建国家5A级旅游景区，城步南山国家公园体制试点区获批。邵东、城步被授予国家电子商务进农村综合示范县，北塔获批湖南省服务业示范集聚区，“洞口农业产业带”在中国惠农网上线运行，雪峰蜜橘、武冈卤菜网上电子交易额屡创新高，达到42亿元。新培育中国驰名商标4件，中国地理标志证明商标5件。引进商业银行5家，完成10家农村商业银行改革。金融机构存款余额由1320亿元增加到2400亿元，贷款余额由525.85亿元增加到1050亿元，存贷比达到43.75%，比2012年提高3.91个百分点。

四年来，我们着力补短板强基础，区位优势日益凸显。累计实施重点项目1251个，完成投资2144.7亿元。城市功能不断强化。中心城市建成区面积由50平方公里增加到72平方公里，人口由55万增加到72万，城镇化率由36.06%提高到43.75%，成功创建省级文明城市和省级园林城市。成功实现中心城区“限摩限电”。高标准拓宽、油化、亮化、绿化、美化了一批城区主街道和邵水东、西路沿江风光带，改造小街小巷367条，新建和改造农贸市场42家、公厕569座，配套完善地下管网791公里，建成城区公共自行车服务系统。城区主街干道清扫保洁全面市场化，环卫机械化作业率达到40%。全民动员，全员参与，你追我赶，夜以继日，强力推进创建国家卫生城市各项工作，一次性顺利通过国家专家组暗访评估和技术评估，各项指标均达到创“国卫”标准。彻底改变了过去“脏乱差”形象，改写了邵阳历史，改变了世人印象，提升了邵阳人民的获得感、幸福感、自豪感。交通状况根本改观。沪昆高铁通车，娄邵铁路扩改竣工并开行动车，怀邵衡铁路进展较快。武冈机场今年通航。洞新、怀通、邵坪、安邵、娄新、衡邵高速相继通车，武靖高速邵阳段即将通车，崀山（白新）高速、呼北高速完成前期工作，全市新增高速公路210公里，总里程达到506公里，排全省第4位，排名前移2位。新改建干线公路887公里、农村公路3016公里。建成农村客运招呼站3440个。改建渡口码头170座，危桥改造及渡改桥173座。能源建设异军突起。投资158亿元，建成和在建风电装机容量60万千瓦，占全省总装机的三分之一，成为全省风电第一市。新一轮农网改造提前一年在全省率先完成。城网建设加快推进，110千伏城西变扩建工程、配电变压器布点项目竣工投产。新建220千伏变电站5座，新建和改造110千伏变电站17座。4G信号覆盖绝大部分行政村。管输天然气正式入邵并向县城推进，市区煤改气工作全面推行。建有沼气池23万多口，新增太阳能路灯10万盏，光伏电站装机容量1.5万千瓦。水利条件持续改善。完成中央财政农田水利项目建设资金4.58亿元。建成农村安全饮水工程929处，解决171万人安全饮水问题。除险加固6座大型病险水闸和1040座病险水库。扎实推进大圳灌区和六都寨灌区续建配套项目建设。治理中小河流河道310公里。治理水土流失面积118平方公里。新增农村水电装机容量3.56万千瓦，新增发电量2.42亿千瓦时。可以说，这四年，城乡基础设施建设突飞猛进，邵阳中心城区“脱胎换骨”、旧貌变新颜！

四年来，我们着力抓改革促开放，发展活力竞相迸发。市本级行政审批项目由301项精简到153项，非行政审批项目由150项精减到43项。市、县政府职能转变和机构改革全面完成。超额完成并乡合村工作任务，减少乡镇30个，合并2275个建制村，超额合并156个村。工商登记制度改革深入推进，实施“五证合一、一照一码”登记制度，市场主体快速增长。供销社、农村综合、文化体制、医药卫生、财税金融、投融资、公车管理等各项改革全面实施。供给侧结构性改革迈出实质性步伐。国企改革、关闭落后小煤矿、关闭烟花爆竹生产企业、不动产统一登记等创造“邵阳经验”，农村卫生室标准化建设创造“武冈经验”。国家公园体制改革、电子商务进农村、依法逐级走访、社保“一卡通”等20余项国家和省级改革试点在我市实施。成功承办2015湖南经济合作洽谈会暨第七届湘商大会和湘台经贸交流合作会，成功举办两届邵商大会，参会邵商游子盛赞家乡巨变。引进落户了华润、恒大、碧桂园、九兴控股、百威英博、亚洲富士电梯等一批知名企业，落户世界500强企业4家，实现零的突破。累计利用外资7.95亿美元，年均增长11.2%，利用内资3419亿元，年均增长24.2%，完成进出口39亿美元，年均增长20.8%。新增境外投资企业110家，总量达到298家。海关商检通关服务中心建设着手启动。我市与澳大利亚怀昂市结为友好城市，实现国际友城零的突破。改革激发活力，开放焕发生机。可以说，这四年，我们攻坚克难，咬紧牙关干成了几件卸包袱、强后劲、生活力的大事实事，邵阳大地到处洋溢着改革开放的气息，820万邵阳人民齐心协力奏响了“春天的故事”！

四年来，我们着力办实事解难题，人民生活不断改善。我们以扶贫攻坚为总揽，着力建机制、补短板、兜底线，民生财政投入持续加大，在财政总支出中占比达71.56%。整合投入各类扶贫资金79亿元，实现77.5万贫困人口脱贫，贫困地区群众收入增幅高于全市平均水平。新增农村劳动力转移就业23.5万人，新增城镇就业19.95万人，城镇登记失业率控制在4.07%以内。市区被征地农民社会保障工作正式实施，覆盖城乡的养老、医疗保险等社会保障体系基本建成。发放低保资金40亿元，医疗救助资金5.2亿元，建有敬老院178所。住房公积金覆盖面持续扩大，累计提取住房公积金22.7亿元，发放个人住房贷款58.7亿元。人均住房保障面积从10平方米提高到13平方米。改造供水管网280公里，新建管网300公里，市本级水质检测能力达到106项。新建江北、洞口、邵东污水处理厂，完成污水管网铺设280公里。新建燃气主干管网620公里，新增管道天然气用户7.06万户。建设公共租赁住房5.89万套、325.3万平方米，发放租赁补贴17万户、1.8亿元。棚户区改造12.5万户，争取上级专项补助资金45.6亿元。完成农村危房改造11.08万户，走在全省前列。农村公路通达工程100%到村。城镇居民人均可支配收入突破2万元大关，由15485元提高到22966元，农民人均可支配收入逼近万元关口，由5136元提高到9745元。106项重点民生实事指标完成或超额完成。关爱留守儿童工作取得实效。

四年来，我们着力强保障促和谐，社会事业全面进步。投入42亿元改善办学条件，学前教育、义务教育、高中教育入学率分别达到78.3%、99.5%、88.7%。实施市城区化解“大班额”三年行动计划，新增学位12960个。邵阳医专并入邵阳学院，成为全省规模最大、学科门类最全的“二本”学校。启动职教新城建设，积极创建湘中幼儿师范高等专科学校。湘郡铭志学校正式建成并招生入学。市一中搬迁着手启动。科技创新能力稳步提升，申请专利6575件，专利授权3852件，高新技术企业达46家，邵阳国家农业科技园区成功获批。新增“三甲”中医医院3所，三级综合医院1所，医疗机构总数达7426个。“单独二孩”、“全面二孩”政策稳步实施，人口自然增长率控制在7.9‰以内。文艺精品创作成果丰硕，祁剧《梦蝶》首次入选国家舞台艺术精品工程，广播电视“村村响”、“村村通”工程基本完成。持续加强生态环境保护，建设自然保护区7个，全市整体纳入首批国家生态文明先行示范区，绥宁、城步、新宁被列入国家重点生态功能区。创建省级生态乡镇93个、生态村100个，隆回虎形山瑶族乡成功创建国家级生态乡镇。全面推行“一村一辅警”建设，全市3299个村全部建立辅警工作站，走在全国前列。严厉打击各类违法犯罪活动，实施“公安消防大建设三年行动计划”，平安邵阳建设全面推进，社会治安持续平稳，综治民调排名逐年进位，由全省最后上升到全省第五位，“邵阳不稳”的旧记忆正逐步淡化褪色。以淘汰高危行业落后产能为抓手，强化安全监管责任，推行监管重心下移，筑牢基层基础，全市煤矿从107对关退至8对，烟花爆竹生产企业整体退出，近四年未发生重特大事故。国防动员和国防教育体系不断完善，双拥共建、优抚安置深入推进，首获“全国双拥模范城”称号。新邵县陈家坊镇被评为“全国民族团结进步示范单位”。投入11亿元，建成1206、1213人防工程。驻邵解放军和武警部队在支持地方建设等方面作出了重要贡献。同时，积极开展统计、审计、人事、编制、食药、质检、档案、移民、民族、宗教、海事、国安、供销、气象、地震、地方志等工作，工会、共青团、妇女儿童、残联、文联、工商联、台湾事务、港澳事务、社会科学、红十字会、无线电管理、驻外办事机构、邮政、通讯、石油、烟草、盐务、铁路、高等院校、科研院所等方面的工作取得新的成绩。

四年来，我们着力转作风优服务，行政效能有效提升。坚持依法行政、从严治政、廉洁勤政，政府自身建设不断加强。自觉接受市委的正确领导，主动向市人大及其常委会报告工作，主动与市政协、各民主党派、无党派人士和工商联协商工作。市人大代表建议和市政协提案办复率100%，满意率99%以上。推进法治政府建设，电子政务和电子监察系统建成运行，政府信息全面推行公开，12345政府服务热线正式开通。认真落实中央八项规定精神和国务院“约法三章”要求，扎实开展党的群众路线教育实践活动、“三严三实”专题教育和“两学一做”学习教育，从严从实整治“四风”、“雁过拔毛”等突出问题，坚定不移推进党风廉政建设和反腐败斗争，政风行风进一步好转。

各位代表，2016年是本届政府的收官之年，我们积极应对经济下行压力，保持定力、尽力作为，办成了一批实事，啃下了一些硬骨头，实现了“十三五”的良好开局。经济运行保持较好态势。11项主要经济指标，10项增速高于全省平均水平，增速排位整体处于全省中上水平。工业发展总体平稳。湘商产业园建设进展迅速，完成固定资产投资34.35亿元，新建标准厂房145万平方米，续建125万平方米，竣工164万平方米，投产110万平方米，签约企业283家，入驻企业147家，投产企业95家。“四百工程”项目新开工148个，竣工投产112个，招商153个，新增规模企业110家。出台降低实体经济企业成本21条硬性措施，对企业各类成本性费负能免的免、能降的降、能减的减。特别是把解决企业“过桥资金”列为帮扶重点，市财政拿出3亿元资金帮助中小企业解决融资难题，其中2亿元用于企业续贷过桥资金，1亿元扩充市中小企业信用担保公司资本金，帮助一批有潜力、有市场的困难企业渡过了难关。城乡建设强力推进。启动实施“城市基础建设三年行动计划”，“两桥四路”等城市骨架工程，市体育中心、市文化艺术中心、幼师高专等重点民生工程，中心城区主干道临街建筑立面“穿衣戴帽”美化工程快速推进。市本级和三区近20个项目2016年底集中开工。以排山倒海之势，持续推进“创国卫”工作，中心城区面貌进一步改观，新城气息更加浓厚。县市创卫步伐紧随其后，隆回、武冈继续保持省级卫生城市称号，绥宁、城步、新宁等县可望创成省级卫生城市。投入1亿元推进“三百”行动，打造了资新、西湖桥、双清、塔北、白洲社区等一批秀美城中村和城乡接合部。新创建省级“美丽乡村”29个、市“美好社区”120个。民生民利持续改善。整合投入各类扶贫资金49.38亿元，实现20.4866万贫困人口脱贫。采取市统贷、县分还、市贴息和“六统一”办法，投入30亿元，为全市建档立卡贫困户中的51126户无房户和D级危房户重建新房，春节前全部搬进新居，一举结束了全市贫困户无房住的历史，一年赶超，走到了全省前列，是全省无房、D级危房贫困户率先实现危改清零的市州。投入45亿元推进棚户区改造，41790户城市棚户区改造、956户国有工矿棚户区改造实现100%开工，货币化安置比例逐步提高。市中心医院在全省市级医院综合实力排名跃升到第一位。启动村级卫生室建设大行动，全年共完成了1050个村级卫生室建设。市区被征地农民社会保障工作有力推进，惠及群众30多万人。安全生产形势平稳，全年未发生较大以上生产安全事故。实施“雷霆行动”，打黑除恶、缉枪治爆、整治新型毒品等工作成效显著，全市治安环境明显好转。

各位代表，四年的工作实践，我们深切体会到：必须始终坚持市委的坚强领导，坚决做到党委有号召、政府有行动，党委有要求、政府有落实，深入贯彻落实市委的决策部署，保持正确的政治立场和发展方向；必须始终高举加快发展旗帜，解决邵阳所有问题的关键在发展、出路在发展，任何时候都要坚持发展第一要务，保持发展定力，一张蓝图绘到底，一个思路贯始终，不断做大经济总量，做强经济存量，做优经济增量，提升发展质量；必须始终弘扬攻坚克难的担当精神，抓住事关发展大局的重点、难点，自我加压、敢打敢拼，每年集中力量办成几件大事实事，力克瓶颈制约，实现重点突破，为经济社会发展扫除关键障碍；必须始终坚持以人民为中心的发展理念。把保障和改善民生贯穿各项工作始终，决策以民生为首选，支出以民生为首位，工作以民生为首要，全力实现好、维护好、发展好最广大人民的根本利益，努力维护公平正义，调动全市人民积极性、主动性和创造性，让人民群众享有更多获得感！

各位代表，四年取得的成绩来之不易，是市委统揽全局、正确领导的结果，是市人大、市政协、邵阳军分区和社会各界鼎力支持的结果，是全市人民戮力同心、拼搏奋进的结果。在此，我代表市人民政府向全市人民和各级干部，向所有关心、支持邵阳发展的同志们、朋友们，表示衷心的感谢，并致以崇高的敬意！

在肯定成绩的同时，我们也清醒认识到存在的问题和不足。主要是：经济总量小，人均水平低，贫困人口多，城乡差距大；工业化、城镇化水平不高，传统产业转型升级慢，战略性新兴产业还比较弱；农业基础比较薄弱，服务业整体水平较低，部分行业企业生产经营困难；2016年项目建设推进较慢，固定资产投资增幅回落，工业经济增速放缓；少数干部不作为、慢作为、乱作为的现象依然存在，发展环境有待进一步优化。我们将正视问题，直面挑战，采取有效措施，切实加以解决，决不辜负各位代表的重托，决不辜负全市人民的厚望！

二、今后五年目标任务

新一届政府任期至2021年，届时，我们将迎来中国共产党成立100周年的光辉时刻，我们将实现第一个百年奋斗目标，全面建成小康社会，进而向第二个百年宏伟目标迈进。这是我们的梦想与荣光，这是我们的使命与担当。这五年将是我市全面小康的决胜期，也是我市挺进全省第一方阵的决战期。国家实施“一带一路”、长江经济带、中部崛起战略，我省着手布局“一带一部”区域发展战略，有利于我市开放崛起，承接沿海产业转移，培育经济发展新动能。中央向贫困宣战，出台一系列“滴灌式”脱贫政策，有利于我市补齐短板、改善发展条件，打赢脱贫攻坚总体战。我市业已形成和正在形成的产业基础、交通条件、生态资源、人口红利和干事作风，为我们加快发展提供了坚实物质基础和宝贵精神财富。尤其是市第十一次党代会提出“二中心一枢纽”战略目标，进一步明确了方向，凝聚了共识，必将激励全市人民积极投身新的伟大实践。我们必须认清形势不犹豫，坚定信心不动摇，咬定发展不松懈，抢抓机遇，奋发有为，推动产业进入中高端，确保发展保持中高速，力争市域总体实力排名进入中高位，让邵阳更有面子、更有位子，让全市父老乡亲生活得更有质量、更有尊严！

未来五年的总体要求是：高举中国特色社会主义伟大旗帜，深入贯彻落实习近平总书记系列重要讲话精神，特别是关于经济工作一系列新理念、新论断、新部署、新要求，统筹推进“五位一体”总体布局，协调推进“四个全面”战略布局，牢固树立“五大发展理念”，全面落实省、市第十一次党代会精神，以“创新引领、开放崛起”战略为指引，坚持稳中求进总基调，做到“稳”得更牢靠，“进”得更有力，着力推进供给侧结构性改革，着力保障和改善民生，着力推进农业现代化，加快构建现代产业体系、新型城镇体系、综合交通枢纽体系，加快建成“二中心一枢纽”，全面建成小康邵阳。

未来五年的工作目标是：经济保持中高速增长，增速高于全省平均水平。到2021年，GDP达到2300亿元，年均增长8%。财政总收入达到230亿元，年均增长10%。规模工业增加值年均增长8%。固定资产投资年均增长15%。社会消费品零售总额年均增长12%。城镇居民人均可支配收入达到36000元左右，农村居民人均可支配收入达到16000元左右，城乡居民人均可支配收入年均增长10%。一半以上县域经济达到或超过全省中等水平，邵东进入全省十强县。现行标准下贫困人口全面脱贫，贫困县贫困村全部摘帽。经济综合实力稳居全省第二方阵首位，力争跻身全省第一方阵。通过全市人民奋力拼搏，努力让邵阳经济更强，生态更好，形象更佳，人民群众的笑容更加舒心而灿烂，努力实现古城邵阳意气风发迈进“发达地区”的追求与梦想！

1。着力打造现代产业新高地，实现经济实力新突破。上世纪七八十年代，邵阳曾经是全省经济大市和工业重镇，工业总产值占全省六分之一（“十一五”末期占全省二十分之一），经济总量排全省第五位，形成了“长衡株潭邵”的历史排名。全国41个工业门类，邵阳就有39个，其中印刷机械、纺织机械、医药化工、液压件、航天工业等支柱产业在全省叫得响、全国有影响。邵阳曾经生产或拥有全省第一辆汽车、第一台计算机、第一台洗衣机、第一台印刷机、第一台纺织机、第一台电风扇、第一支钢笔、第一包洗衣粉，中国第一个民营经济试点县在邵阳，湖南第一个财政亿元县在邵阳，邵阳的工业有过辉煌的历史。由于种种原因，邵阳经济特别是工业在全省排位后移，直到“十二五”我们才再次扬起逆势上扬、赶超进位的风帆。邵阳要加快赶超，跻身“发达地区”行列，出路在工业，第一推动力靠工业。未来五年，工业发展咬定冲刺“百亿企业、千亿产业、国家级园区”目标。加快以邵阳经济开发区为核、沪昆高速百里工业走廊为带、县市工业园区为点的“一核一带多点”建设，努力打造湘西南规模最大、产能最足、后劲最强、品位最高的“工业重镇”。落实《中国制造2025》要求，力促智能制造、新兴材料、环保技术、新能源新动力、网络信息、民用航空等一批高新项目签约落地、建成投产，努力在高端装备制造和现代信息产业上实现新突破、大跨越。依靠创新引领、科技支撑，打造传统产业升级版，皮革、箱包、小五金、打火机和食品等传统优势产业提品质、创品牌。通过整合、引进、嫁接，发制品由收集清洗、原料出口升级为品牌加工、出口贸易，形成百亿产业群。隆回金银花产业链产值突破百亿元。支持邵阳县建立中国南方油茶交易中心，形成百亿油茶产业链。廉桥药材市场跻身全国十强。打造景观，改善设施，发展全域旅游。全力扶持实体经济，推动大众创业、万众创新。树立强烈的质量意识，深入开展“增品种、提品质、创品牌”专项行动，发扬“工匠精神”，培育“百年老店”，增强产品和服务竞争力。坚持信息化带动工业化，促进两化深度融合，实现邵阳工业化初期向工业化中期历史性飞跃，努力实现二产业在三次产业中的占比领先一、三产业，以新型工业化领跑全市经济发展。通过五年或更长一点时间的苦干实干，努力实现邵阳工业重振雄风、再造辉煌，努力为邵阳人民争气，为古老宝庆争光！

2。努力建设人民满意城市，实现城镇建设新突破。实施中心城区扩容提质，市中心城区建成面积100平方公里、人口110万的“双百”Ⅱ型大城市，成为湘中湘西南唯一过百万的大城市。做大做强东部城镇群，东部四县城区人口达到120万，重点镇人口100万，东部城市群成为城镇人口330万、辐射500多万人的湘中湘西南最大城市群，形成省域副中心。支持邵东撤县建市。做优做美西部生态圈，以武冈市区为中心，以西部县城为支点，形成点轴发展。支持武冈建设市域副中心，扶持新宁、绥宁、城步等西部县加快发展。加快县城建设，县县争创省级卫生城市，有条件的创建国家卫生城市，县县建成文体中心，县县建设一批城市休闲公园和景观小品，县县实施旧城改造，县县城区主街干道完成“白改黑”。努力打造一批新型旅游名镇、工贸强镇、文化古镇、省际边界重镇和全国重点镇。以“数字城管”系统为载体，形成运作市场化、管理网格化、智能化、信息化、作业精细化、考核标准化的长效城管体系。合理布局城市公共设施，完善公共服务。推动城乡统筹发展，基础设施向农村集镇延伸，推进城乡一体化。坚持生态优先，形成绿色低碳生产生活方式和城市建设运营模式，大力开展生态修复，努力打造天蓝、地绿、水净的美好家园，让全市人民生活更舒心，工作更称心，办事更顺心，全社会更有爱心，对未来更有信心！

3。加强城乡基础设施建设，实现区位条件新突破。交通，按照建设区域性综合交通枢纽目标，实现大跨度、全覆盖、高密度综合发达的铁、公、机无缝对接，构筑以沪昆高铁、呼南高铁为基础的“三纵三横二连”快速铁路米字型结构，以沪昆高速、二广高速和呼北高速为基础的“三纵三横两连”高速公路网状型结构，以“一东一西”两支线机场为核心、多个通用航空机场为点的航空网络。开通东城快线、西城快线，完成城市三环线及地下综合管廊建设。怀邵衡铁路、邵阳火车站综合客运枢纽和邵阳东货场开通营运，开工建设呼南高铁邵永段，争取兴永郴赣铁路邵阳段开工，谋划推进张崀桂铁路、邵冷铁路和邵东至市区至武冈城市轨道交通前期工作。建成崀山高速，开工建设呼北高速隆回金石桥至武冈段，争取城步至广西龙胜高速开工，争取洞口至溆浦高速公路纳入湖南省高速公路网规划。水利，加快推进衡邵干旱走廊综合治理、娄邵盆地基本农田建设和农业综合开发高标准农田建设，完成2处大型灌区和48处中型灌区续建配套与节水改造工程。加快完善市、县城区防洪堤及沿江风光带建设，继续实施病险水库及水闸除险加固和五小水利工程，打通防汛抗旱最后一公里。实施河、湖、湿地互流互通。推进城市第二水源建设，实施55处农村水电增效扩容改造、50处小水电扶贫项目。能源，实施城网建设三年行动计划，推进智能电网建设。加快新能源基地建设，稳步发展水电，壮大风电，发展光伏和生物质能发电。完成“气化邵阳”建设。信息化，加快“三网融合”，推进电子政务、电子商务、电子教育、电子金融、电子社区发展，提高公共服务领域的数字化、网络化以及社区、家庭的信息化水平，推进宽带网络从乡镇向行政村、自然村延伸。大力发展互联网+，助力经济发展。通过基础设施大投入、大建设、大改造、大提升、大跨越，彻底甩掉“落后地区”的帽子，昂首阔步迈向湖南“发达地区”行列！

4。聚焦聚力改革开放，实现发展动能新突破。面对经济“速度换挡节点”、“结构调整节点”、“动力转换节点”，以满足需求为最终目的、提高供给质量为主攻方向、深化改革为根本途径，狠下决心推进供给侧结构性改革，落实“三去一降一补”任务，着力形成新供给，释放新需求，培育经济增长新机制新动能。突出减税、清费、降成本，强化“放水养鱼”意识，落实降低实体经济成本实施方案和物流业降本增效专项行动，提高“放管服”质量，大力降低制度性交易成本。通过债转股、股权融资等方式降低企业债务，提高资产收益率，切实把企业杠杆率降下来。打通去房地产库存和促进人口城镇化、棚户区改造结合通道，加大棚改货币化安置力度。深化财税体制改革，建立现代财政制度。深化市场配置要素改革，推动公共资源配置市场化，加快建立现代产权制度，构建统一开放、竞争有序的现代市场体系。推进自然资源管理制度改革，全面落实不动产统一登记制度。推进混合所有制改革，鼓励、支持、引导非公有制经济发展。积极推进金融改革，健全金融市场体系，创新融资模式和融资渠道，加快政府融资平台市场化转型，防范化解金融债务风险。密切银政企对接，扩大金融供给，破解企业融资难、融资贵难题。推进农业供给侧结构性改革，抓好现代农业产业园、科技园、创业园建设，推动农业农村发展由主要满足“量”的需求向注重满足“质”的需求转变，促进农业经济调结构、转方式、提质量、创品牌、增效益。坚持开放带动、创新驱动、统筹推动、“三外”联动，加快发展开放型经济。加快承接产业转移示范区建设，打造经济发展新的增长极。加强邵阳发制品、邵东打火机和箱包、新宁隆回裘革、武冈洞口邵阳县生猪等基地建设，完善品牌建设培育体系，培育一批年进出口额过1亿美元的“出口生产基地”和年进出口额过10亿美元的产业。引导本土企业通过设立境外公司，拓展销售渠道，建立直销平台。加快构建海关、商检平台，建成市进出口通关服务中心，引进培育一批跨境电子商务平台和企业，推进“单一窗口”和大通关建设。改革开放无止境，我们再不能输在新一轮改革开放的起跑线上。我们要解放思想，更新观念，革故鼎新，跟上时代，以抓铁有痕的决心、壮士断腕的勇气，向封闭守旧的陈腐观念宣战，向束缚发展的体制机制开战，驱除“拦路虎”，踢开“绊脚石”，向深化改革要动力，向创新引领要活力，向开放崛起要红利，让古城宝庆沐浴改革开放的浩荡东风，焕发勃勃生机！

5。全面协调推进社会事业，实现共建共享新突破。坚持把脱贫攻坚作为重大政治任务和第一民生工程，坚决打赢脱贫攻坚战，决不让一个贫困地区、一个贫困群众掉队，贫困县、贫困村全部脱贫摘帽，现行标准下贫困人口全部脱贫。加快普及高中教育，加强就业服务、社会保障、基本医疗和公共卫生、公共文化、环境保护等基本公共服务，建立更加公平更可持续的社会保障制度。规划20平方公里，迁建新建、整合重建24所职业院校，建设湘中湘西南最大的职教新城。推进保障性住房建设，完成棚户区改造任务，建立公开规范的住房公积金制度，加快住房公积金向农民工和个体工商户延伸。大力促进创业就业，持续增加城乡居民收入。积极创建国家健康城市，广泛开展全民健身运动，建成市体育中心，当好东道主，高质量高水平办好第十三届省运会。加快推进公立医院改革步伐，加强乡镇卫生院、社区卫生服务中心和村卫生室建设，基本建成城乡居民15—30分钟医疗服务圈，解决人民群众看病难、看病贵问题。做好人口计生服务工作。加大科技研发投入，创建国家级技术研发中心和省级“星创天地”，争取科技成果转化80项，发明专利申请量和授权量年均增长12%以上。加强社会治安综合治理和突发公共事件处理，积极化解社会矛盾，努力构建平安邵阳。落实安全生产责任制度，坚决遏制重特大安全事故发生。继续淡化人们过去对邵阳治安稳定方面的不良记忆，努力传播邵阳文明有礼的正能量，让世人真真切切地感受到：邵阳这片土地从来不甘于平庸，邵阳这方人民从来不惧怕困难；让世人真真切切地感受到：“心忧天下”是邵阳人民的情怀，“敢为人先”是邵阳人民的胆识，“经世致用”是邵阳人民的智慧，“坚韧不拔”是邵阳人民的意志，邵商行天下，天下邵阳人都是好样的！

三、2017年工作

2017年是新一届政府的开局之年，也是全面贯彻落实省、市第十一次党代会精神、加快实施“十三五”规划的重要一年，我们将迎来党的第十九次代表大会胜利召开，做好今年的工作意义重大。2017年，经济社会发展预期目标是：GDP增长8.5%，规模工业增加值增长8%以上，固定资产投资增长16%左右，财政总收入增长8%左右，社会消费品零售总额增长12%，利用外资增长10%，实际利用内资增长15%，进出口总额增长10%，城乡居民人均可支配收入增长10.5%，人口自然增长率控制在9‰以内，居民消费价格涨幅保持在3%左右，万元GDP能耗下降3%。以稳中求进的坚实步伐、实干兴市的优异成绩向党的第十九次代表大会献礼！

围绕上述发展目标，突出抓好五个方面的工作：

1。坚持“四跟四走”路子，全力推进精准脱贫。大力实施交通、水利、电力和光伏、农危改造和环境整治、农村“互联网＋”、文化、乡村旅游“七大扶贫工程”，全面推进特色产业、劳务输出、易地扶贫搬迁、生态补偿、教育扶贫、医疗救助、保障兜底“七大扶贫行动”。市财政拿出7000万元资金，奖补“六个一批”。整合各类资金5亿元，一年内全部解决贫困人口的自来水问题。整合各类产业扶贫资金，集中投向产业扶贫项目，通过龙头企业、产业能人和各类农业合作社引领带动，做实产业扶贫。加大电商扶贫、光伏扶贫、金融扶贫力度，对贫困户在“公司+农户”运作模式的公司4万元以内的入股经营，给予可申请50%的免抵押贷款政策支持。加大旅游扶贫力度，大力开发乡村特色旅游，帮助农民增收。有序引导、培训贫困农民就近转移就业、增收脱贫。发挥市财政投入面上村1100万元专项扶贫资金的杠杆效应，用好用足贫困户每户5万元扶贫贷款，通过直接帮扶、委托帮扶、股份合作等方式，让贫困户有产业、有股红、有活干、有收入。落实教育扶贫政策，尽可能多地把贫困户中的适龄孩子送进职业院校，将来能有稳定的就业、不错的收入和体面的生活，从而斩断贫困代际传递。抓好兜底保障工作，建立兜底保障动态数据库，及时通过“一卡通”发放补助资金。全年减少贫困人口25万，538个贫困村脱贫摘帽，面上县区贫困人口全部脱贫，贫困地区农民人均可支配收入增幅保持在全市平均水平以上。

脱贫攻坚是重活、难活，更是实活、细活。我们要用心、用力、用感情，有招、有为、有办法，真脱贫、脱真贫，绝不能数字脱贫、假脱贫。在脱贫问题上，凡是无动于衷、无所作为的，我们都要断喝一声、猛击一掌；凡是欺上瞒下、弄虚作假的，一律严肃查处、严格问责！

2。把投资作为稳增长的关键，全力推进项目建设。尽快把2016年未开工项目开起来，力争2017年新开工重大项目80个。各县市陆续启动县城提质“十个一”项目。实施300个重点建设项目，年度投资700亿元以上。铁路，怀邵衡铁路确保2018年上半年、力争2017年竣工通车。全面启动邵阳高铁（南）站综合交通枢纽建设。争取呼南高铁邵永段完成可研批复等前期工作，2018年正式开工。高速，做好城步至龙胜高速开工前期工作，加快建设武靖、新桂高速，开工建设崀山高速，完成邵坪、安邵高速扫尾后续工程，完成呼北高速隆回至武冈段前期工作。站场，建成邵东灵光殿、新宁回龙寺、洞口黄桥旅游客运站，加快邵阳坪上综合客运枢纽建设，开工建设邵东汽车东站。加快推进干线公路、农村公路安保工程、危桥改造、渡改桥项目建设，启动三区115.8公里国省干线公路、542.6公里农村公路改造升级建设。能源，完成全部贫困村的农网改造升级，加快“邵阳-隆回-洞口-武冈-新宁”输气管线建设。水利，加快防洪闭合圈建设，新建堤防13.2公里。启动木瓜山第二水源工程，做好神滩提灌工程前期工作。狠抓五小水利工程建设，全面硬化灌区325公里骨干、支渠，整治塘坝4000口。

3。以产业建设为龙头，不断壮大工贸实力。做大做强工业产业。继续抓好“四百工程”，全年新增规模工业企业110家，工业固定资产投资和技改投资均增长10%以上。食品医药产业重点抓好百威啤酒、统一企业、湘中制药、湘窖二期等项目，装备制造产业重点抓好富士电梯、通达汽零、兴达精密机械、三一智能渣土车、邵阳纺织机械等项目，轻工产业重点抓好合隆国际羽绒制品、发制品园、立得皮革皮具制品等项目，建材产业重点抓好云峰水泥余热发电等项目。以上这些项目要倒排工期，按时序节点推进，确保2017年基本建成投产。对最近陆续洽谈靠岸的几个战略性新兴产业项目，明确专门领导，组建专责班子，盯牢跟紧，尽快签约落地，尽快开工建设，争取早日建成达产。湘商产业园续建竣工标准厂房110万平方米，沪昆百里工业走廊沿线园区新建标准厂房60万平方米，新增入园企业150家，建成标准厂房使用率不低于70%。力争园区70%以上的产业项目投产，新增工业总产值260亿元，税收10亿元。加快申报国家级经济技术开发区和国家级创新创业示范基地。加快推进农业现代化。围绕扩大中高端有效供给，深化农业供给侧结构性改革。大力推进农业农村三个“百千万”工程，抓实“四边五年”绿色行动。抓住我市农村住房正处于新一轮改造升级高峰期的机遇，加强统筹协调和配套建设，打造一批美丽乡村，改善农村生产生活环境。试行“三权分置”，推动农村产权流转交易。培植龙头企业、种养大户和各类农业专业合作社，大力发展休闲农业、观光农业、扶贫农业，通过农业产业化、农产品销售电商化带动农民致富。积极发展现代服务业。大力发展金融业。加大银政企有效对接，努力提高存贷比。充分利用国家为贫困县企业IPO开辟绿色通道的有利条件，争取实现邵阳企业主板上市零的突破。创新农村金融产品，提高农业保险服务覆盖面。大力发展物流业，加快推进物流中心建设，加快培育出口基地和出口品牌。实施“互联网+商贸流通”行动计划，加快建设电商产业园、电子商务示范企业、第三方电子商务平台和公共服务平台，引进大型电商入驻邵阳。实施市城区重点旅游景区建设三年行动计划，推进大崀山旅游功能区建设，加快恢复宝庆古八景、建设新八景、修筑八大文化场馆、打造资江八桥景观，启动北塔生态文化旅游园建设。加快南山4A级景区创建工作。建设一批精品旅游小镇、美丽乡村、古寨名村。总之，要通过上项目、提品质，把产业做大，把工贸做强，把景点做美，把商旅做活，从而推动邵阳经济更有后劲，更有品质，更具活力，更有动能和实力！

4。加快新型城镇化进程，充分挖掘内需潜力。开工建设城区三环线及地下综合管廊。加快改造城中村、城乡接合部。学院路二期（塘邵快线）建成通车。启动东城快线建设，加快西城快线前期工作，打通中心城区南北通道，尽快构筑起“十字架”快速通道。“两桥四路”、新华南路二期建成通车。加快推进新城大道、邵西大道、魏源西路、湖口井路、邵檀路、邵水西路南段、邵水东路南段和宝庆新城、江北新城路网建设。围绕中驰国际广场等9个棚户区改造示范项目，打通迎春路、电机路、建设北路等瓶颈路段。择优培育和发展小城镇，抓好廉桥、高沙、武阳、陈家坊、六都寨、邓家铺、下花桥等中心镇开发建设。开工建设人防疏散基地紫霞公园，建设一批城区主题公园、小游园、专业花卉园，打造一批星级生态农庄、秀美村庄、花果园。推进污染治理和减排工作，加快推进区域环境综合治理和提质改造。加强大气污染防治，改善城区空气质量。严格管控建筑工地、渣土运输、道路扬尘、露天开采等扬尘污染，建成机动车尾气检测站，财政补贴8000万元，加快淘汰黄标车。加强资江、邵水等重点流域的水污染综合整治，加强饮用水源地保护，切实保障饮用水安全。加强对工矿企业的环境监管。加快推进龙须塘老工业区综合治理及棚户区改造工程，完成洋溪桥污水处理厂二厂主体工程建设。抓好农村环境卫生综合整治，形成一批美丽乡村建设示范带。推进自来水、液化气、环卫基础设施向农村延伸。

5。切实保障和改善民生，加强社会建设。办好民生实事。高标准高质量完成“精准扶贫、农村公路建设、新增城镇就业、棚户区改造、农村适龄妇女‘两癌’免费检查、新增养老床位、新增社会治安视频监控摄像头、农村宽带网络升级改造、行政村配电网改造、产前免费筛查”等重点民生实事任务。投入11亿元，实施公安消防大建设三年行动，力争二年完成。查实做实农村人安饮水工程，推进巩固提升。推广武冈经验，2017年全市统一行动，整合筹集8亿元资金，年内每一个行政村都建成一个标准化卫生室。发放住房公积金贷款24亿元。实施城市棚户区改造项目93个、4.6万户、456万平方米。新建燃气管网30公里，改造燃气管网20公里。新增居民燃气用户1.5万户。推进科教文卫体事业。改善农村学校办学条件，加快解决城区“大班额”问题。投资30亿元，全面启动职教新城建设，谋划打造高品位航空职教区。开工建设市一中，加快湘中幼儿师范高等专科学校建设，确保2017年秋季正式招生入学。做好文保申报工作，推动绥宁侗族村寨申报世界文化遗产。市体育中心主体工程基本完工，精心筹备第十三届省运会。研发经费支出占GDP比重提高0.25个百分点，发明专利申请量、拥有量和专利总量增长12%。完善县、乡、村三级公共卫生管理体制。建成市中心医院东院一期、邵阳学院附属医院住院综合楼并投入使用，开工建设市二人民医院整体搬迁工程。切实维护安全稳定。实施建筑施工、道路交通、特种设备、消防、危险化学品五大领域专项整治，确保安全生产形势平稳有序。8家保留煤矿整体关闭退出。着力构建社会治安立体化防控体系，依法惩治各类违法犯罪活动，增强人民群众安全感。加大禁毒工作力度，做好社区矫正和法律援助工作，全面发挥“一村一辅警”作用，完善社会矛盾排查预警和调处化解机制。让邵阳的发展环境更优，人民群众的安全感更强，让民主法制、公平正义、平安和谐的阳光洒满邵阳大地、普照百姓心房！

各位代表，做好经济社会发展各项工作，必须加强政府自身建设。我们将坚持担当作为，建设实干政府。对事关发展的大事、难事、要事，敢于担当，主动作为；对工作中定下来的事，不折不扣，迅速落实；对项目落地、企业需求、群众困难，不推不拖，限时解决；对市委、市政府的决策部署，加强检查，监督落实，见人、见事、见结果。严格落实“三个区分开来”，建立容错纠错机制，宽容干部在工作中特别是改革创新中的失误，旗帜鲜明地为敢于担当的干部担当，为敢于负责的干部负责！坚持科学施政，建设效能政府。加强公务员行政能力培训，解决不懂做、不会做、不想做、不敢做的问题，进一步提高执行力和推动力。严格落实首问负责、一次性告知和限时办结制度，提高工作效率。发挥政务服务中心、网上电子政务作用，进一步提高行政效能，为群众提供便捷优质的服务。严肃查处不作为、乱作为、慢作为和扰乱市场经济秩序行为，推进诚信邵阳建设，切实优化经济发展环境，降低综合营商成本。坚持依法行政，建设法治政府。依法全面履行政府职能，法定职责必须为、法无授权不可为。完善公众参与、专家论证、风险评估、合法性审查、集体讨论的科学决策机制，完善政府法律顾问制度。依法接受市人大及其常委会法律监督、工作监督，自觉接受市政协民主监督，主动加强与市政协的民主协商，广泛听取和采纳各民主党派、工商联和各人民团体、无党派人士的意见建议。全面推进政务公开，让政府运行更加科学规范、阳光透明。坚持从严治政，建设廉洁政府。严格执行党风廉政建设责任制，自觉践行为民宗旨，坚持不懈改“四风”。强化审计监督，加强对政府投资的重大项目、公共资金、公共资源、国有资产的监管，依法依规依程序推进政府采购、项目招投标、土地招拍挂等活动，严肃查处各类违法违纪行为。厉行勤俭节约，“三公”经费只减不增，最大限度降低行政成本，把更多的钱用在民生实事上。

各位代表，拼搏成就梦想，奋斗赢得未来。让我们紧密团结在以习近平同志为核心的党中央周围，在中共邵阳市委的坚强领导下，凝心聚力，扎实工作，为实现“二中心一枢纽”目标、全面建成小康邵阳努力拼搏，为中国共产党成立一百周年交上一份满意的答卷！
